# A randomized, placebo-controlled trial to determine the course of aminotransferase elevation during prolonged acetaminophen administration

**DOI:** 10.1186/2050-6511-15-39

**Published:** 2014-07-22

**Authors:** Kennon Heard, Jody L Green, Victoria Anderson, Becki Bucher-Bartelson, Richard C Dart

**Affiliations:** 1 Rocky Mountain Poison and Drug Center, Denver Health, Denver, CO, USA; 2 University of Colorado Department of Emergency Medicine, Aurora, CO, USA; 3 Vanderbilt School of Nursing, Nashville, TN, USA; 4 Colorado School of Public Health, Aurora, CO, USA

**Keywords:** Alanine aminotransferase, Drug-induced liver injury, Hepatotoxicity

## Abstract

**Background:**

Acetaminophen administration for more than 4 days causes aminotransferase elevation in some subjects. The objective of this randomized, placebo-controlled trial is to describe the course of alanine aminotransferase (ALT) elevation in subjects administered 4 g/day of acetaminophen for at least 16 days.

**Methods:**

A randomized, placebo controlled trial of acetaminophen (4 g/day) vs placebo. Subjects were healthy volunteers with normal liver enzymes. The primary outcome was the course of ALT during acetaminophen administration. All subjects were treated for a minimum of 16 days. Subjects with ALT elevation at day 16 were continued on treatment until these elevations resolved up to a maximum of 40 days. Subjects were also evaluated for elevation of INR or serum bilirubin as evidence of hepatic dysfunction.

**Results:**

157/205 (77%) completed acetaminophen subjects had no ALT elevation or transient elevations that resolved by day 16. Of the 48 subjects who had ALT elevations at study day 16, 47 continued on acetaminophen and had resolution by study day 40. One acetaminophen subject did not have resolution by study day 40, and the course of aminotransferase elevation suggests an alternative cause. One placebo subject had an ALT elevation at day 16 that resolved by day 22. The highest observed ALT among all acetaminophen subjects was 191 IU/L. The mean ALT at day 16 was 4.4 IU/L higher for the acetaminophen than for the placebo group. No subject developed liver dysfunction.

**Conclusions:**

A minority of subjects treated with 4 g/day of acetaminophen for 16 days will have low-grade aminotransferase elevations that are not accompanied by liver dysfunction and resolve if administration is continued.

**Trials registration:**

Clintrials.gov NCT00743093 registered August 26, 2008

## Background

Acetaminophen (paracetamol) is a commonly used analgesic and antipyretic that has an excellent safety profile when taken in doses of 4 g/day or less [1]. However, several studies have found that a significant percentage of subjects who ingest 2–4 g of acetaminophen per day for more than 4 consecutive days develop asymptomatic elevation of serum alanine aminotransferase (ALT) activity [2-6]. These studies generally stopped dosing before the ALT elevations resolved. Multiple clinical studies have not identified any cases of liver failure with long-term acetaminophen use [7-11]. In contrast to prospective studies that found no cases of liver failure, there are anecdotal reports of patients who develop liver failure while allegedly taking therapeutic doses [12]. Given the widespread use of acetaminophen, if asymptomatic elevations of aminotransferase activity with therapeutic doses can progress to liver failure, even rarely, there is a substantial risk to public health.

The course of these aminotransferase activity elevations when acetaminophen is administered longer than 4 consecutive days is not well characterized. Anecdotally, patients take acetaminophen for years (as single ingredient and in combination with opioids) without symptomatic liver injury. In a small, placebo controlled 4-week study measuring the effect of acetaminophen on warfarin-induced anticoagulation, Parra reported a dose-dependent elevation in mean serum aminotransferase activity in subjects at two weeks during treatment with 2 or 4 g/day of acetaminophen [5]. Unlike other studies, acetaminophen was continued despite the mild increases in aminotransferase activity. These elevations resolved by week 4 of treatment and no subject had evidence of liver dysfunction. However, this study only included 15 acetaminophen-treated subjects and serum aminotransferase activity was only measured at baseline, 2 and 4 weeks. While this study suggests that the aminotransferase elevations resolve if therapy is continued, the course is not fully characterized.

The objective of this study is to describe the changes in serum aminotransferase activity in healthy subjects taking 4 g/day of acetaminophen for a minimum of 16 days and continued until any observed ALT elevations resolve. Our secondary aims were to determine if subjects who had aminotransferase elevations developed liver dysfunction and finally to determine if subjects who develop these elevations share any obvious demographic or clinical characteristics.

## Methods

### Trial design

This was a randomized, placebo controlled trial. Subjects were randomized 4:1 acetaminophen to placebo. This randomization plan was intended to increase the power of the study to detect infrequent adverse events in the acetaminophen group. This study was registered with clinicaltrials.gov (NCT00743093) and approved by The Colorado Multiple Institutional Review Board.

### Participants

This was an outpatient study; subjects were evaluated at one of two clinical research sites. Subjects were recruited from a research listserv, posters, classified advertisement website (Craigslist) postings and media advertising. All subjects provided informed consent.

We included male and female subjects who were age 18 years or older and who did not have any of the following exclusion criteria: History of acetaminophen ingestion on any of the four days preceding study enrollment or a measurable serum acetaminophen concentration at time of enrollment; laboratory testing suggesting active viral hepatitis A,B or C infection; any of the following tests greater than the upper limit of normal at screening: serum aminotransferase or total bilirubin, International Normalized Ratio (INR) or alkaline phosphatase activity; platelet count less than 125,000/mL; positive pregnancy test; history of cholelithiasis (without cholecystectomy); history of heavy ethanol use defined as consuming more than an average of 3 alcohol containing drinks daily or 3 or more alcohol containing drinks on any given day over the preceding 2 weeks prior to study enrollment [13]; new prescription medication started within the previous 30 days; taking isoniazid or warfarin; currently has anorexia nervosa or reports a fasting type diet; clinically intoxicated, psychiatrically impaired or unable to give informed consent for any reason; known hypersensitivity or allergy to acetaminophen.

### Interventions

Subjects were administered acetaminophen 4 g/day or placebo. Subjects were instructed to take 2 × 500 mg tablets every 4 hours for 4 doses each day (the exact timing varied but subjects were asked to take the doses every 4 hours after the first dose each day). The subjects noted the time of ingestion for each dose in a study diary. They also recorded other medications and alcohol consumption. Compliance was verified by study diary and pill counts at each study visit.

We included a placebo group for two reasons. First we recognized that there is day-to-day variation in aminotransferase activity and we felt it was important to be able to account for this variation as we quantified the effect of acetaminophen. Second, we have planned secondary studies to identify potential characteristics and biomarkers that may suggest a mechanism for these elevations.

**Figure 1:**
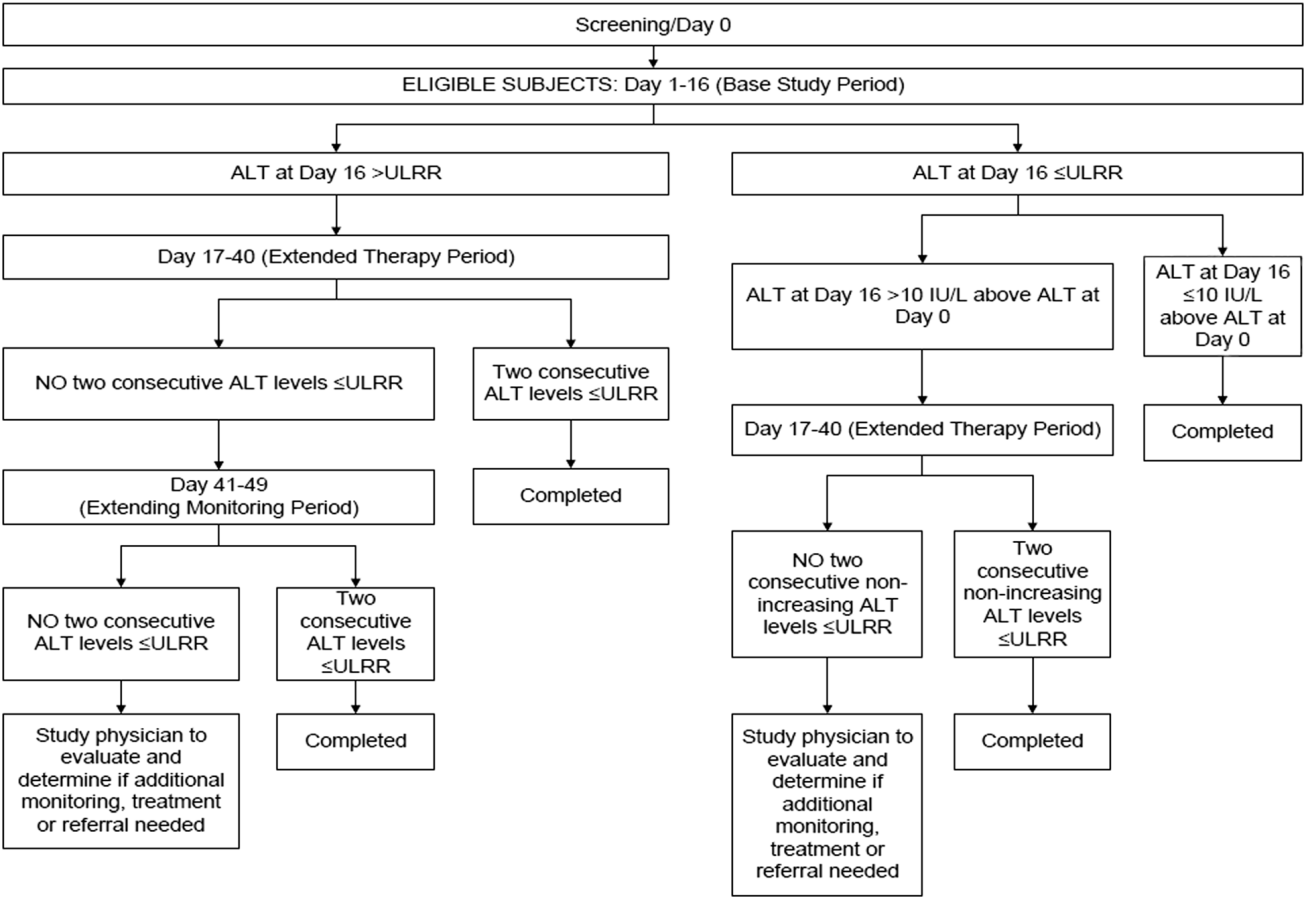
Subject flow and study decision points.

The following tests were performed at each visit: serum aminotransferase (alanine aminotransferase-ALT and aspartate aminotransferase-AST), total bilirubin (TBL) and International Normalized Ratio (INR). Additional testing at the screening visit (study day -7 to -1) included a complete blood count, hepatitis A and C antibodies, hepatitis B antigen, serum acetaminophen and serum pregnancy (females only). Serum acetaminophen concentrations were also measured at day 10 and 16 for safety purposes but were not reported to study personnel unless they were above the critical concentration for our laboratory (>40 mcg/mL or 264 micromol/L) to maintain blinding. Adverse events were recorded using a structured interview at each visit. Subjects were specifically asked if they had nausea, vomiting, abdominal pain or jaundice and asked if they had any other symptoms.Patients were evaluated (including laboratory testing as described above) on study days 0 (baseline), 4, 7, 10, 13 and 16. At day 16, subjects were assessed for resolution (as defined in the outcome section below). Subjects who did not meet resolution criteria by day 16 went into the extended dosing period. In the extended study period, subjects continued treatment as assigned and continued to be evaluated every 3 days until study day 40. If the subject did not have resolution by study day 40, the study drug administration was stopped and the subject was monitored every 3 days until day 49. If the subject did not have resolution by that point, they were referred to a hepatologist for further evaluation and monitoring (Figure 1).

### Outcomes

As ALT is considered a more specific biomarker for the liver than AST, the primary outcome of this study was resolution of ALT elevation. Resolution criteria were defined at day 16 as an ALT that was less than 47 IU/L (the upper limit of the reference range for our laboratory) and within 10 IU/L of the subject’s baseline value. Subjects who did not meet resolution criteria were continued on the study protocol.

The resolution criteria after day 16 were dependent on the day 16 ALT values.

If the subject had a day 16 ALT >47 IU/L, we required 2 consecutive ALT values less than or equal to 47 IU/L for resolution. If the day 16 ALT was <47 IU/L but >10 IU/L above baseline, resolution occurred when they had 2 consecutive non-increasing ALT measurements less than or equal to 47 IU/L (however they could have a final ALT more than 10 IU/L above their baseline, Figure 1). Our secondary outcome was the proportion of subjects who developed liver dysfunction defined by elevation of the INR above 1.4 or serum bilirubin >2× baseline [14].

As the primary study objective was to document the course of ALT while taking 4 gram per day of acetaminophen, our a-priori analysis plan was to restrict outcome analysis to subjects who completed the full protocol (i.e. met resolution criteria or reached study day 40). For safety reasons, subjects who withdrew from the study were required to have at least one measurement of ALT off study drug.

To assure subject safety, subjects were withdrawn if they developed drug induced liver injury as defined by the United States Food and Drug Administration Drug-Induced Liver Injury Premarketing Clinical Evaluation Guidelines [14]: ALT or AST >8× the upper limit of normal (ULN); ALT or AST >5× ULN for more than 2 weeks, ALT or AST >3× ULN and (total bilirubin >2× ULN or INR >1.5); ALT or AST >3× ULN with the appearance of worsening of fatigue, nausea, vomiting, right upper quadrant pain or tenderness, fever, rash, or eosinophilia.

### Sample size

As our primary purpose was to describe the course of ALT changes there was no obvious endpoint to use for power calculations. Therefore, we elected to power our study to measure the proportion of patients who did not meet resolution criteria by study day 40. We selected 40 days as a small previous study had suggested that many patients resolve by day 28 [5]. We determined that a sample size of 300 completed subjects (240 acetaminophen: 60 placebo) was appropriate for our endpoint. This assumes that no subjects in the acetaminophen group would have ALT elevations that did not resolve by day 40. Zero out of 240 subjects would result in an upper 95% confidence limit for probability that the true incidence of non-resolution would be less than 0.015 (less than 1.5% of subjects would not resolve by day 40). We assumed a 15% screen failure and a 15% dropout rate.

### Randomization

Sequence generation was performed using pre-numbered packages dispensed according to a computer generated randomization list. We used block randomization within the following strata: Hispanic females, Hispanic males, non-Hispanic females, and non-Hispanic males. A separate randomized list of assignments was created for each site based on these strata. We stratified by Hispanic ethnicity as a previous study has suggested that the response to acetaminophen differs between Hispanic and non-Hispanic subjects [6].

### Allocation concealment mechanism

Placebo and acetaminophen tablets were provided by McNeil Consumer Healthcare and were identical in appearance. Study medications were packaged into 1-week supplies by a researcher not involved in subject management or data collection.

### Implementation

Tablets were dispensed during a study visit as a 1-week supply and the doses for each day were provided in a labeled packet.

### Blinding

Participants, investigators and statisticians were all blinded to the treatment groups during the main treatment period (days 0–16). We did not assess the success of blinding. When a subject met the criteria to stop at day 16, the treatment group assignment was unblinded to allow recruitment of acetaminophen-treated subjects into a follow-up study. This occurred after completion of the final study visit (including adverse event recording). The physician assessing relatedness of adverse events and the statistician performing the analysis remained blinded to subject treatment group until the study database was locked.

### Statistical methods

Demographic characteristics are presented as medians and interquartile ranges for continuous variables and proportions for categorical variables.

For our primary outcome analysis, we calculated a 95% confidence interval on the proportion of subjects in the active treatment arm with unresolved, persistent elevation of ALT. To determine if ALT elevations were accompanied by changes in liver function (secondary analysis), we calculated a 95% confidence interval on the proportion of subjects in the active treatment arm with elevations of bilirubin or INR.

In a pre-planned exploratory analysis, we analyzed potential associations between aminotransferase elevations and demographic and clinical characteristics. We used logistic regression (PROC LOGISTIC) to determine if any of the following characteristics were associated with our 3 secondary outcomes: age, sex, race/ethnicity (Caucasian, Black, Hispanic, other), baseline ALT, body mass index (BMI) and average daily alcohol consumption (number of 14 gm servings of ethanol per day averaged over the study duration). The 3 outcomes of interest (defined above) were having an ALT elevation more than 1.5 times baseline, having an ALT elevation more than 10 IU/L above baseline, or meeting extension criteria at study day 16.

## Results

**Figure 2:**
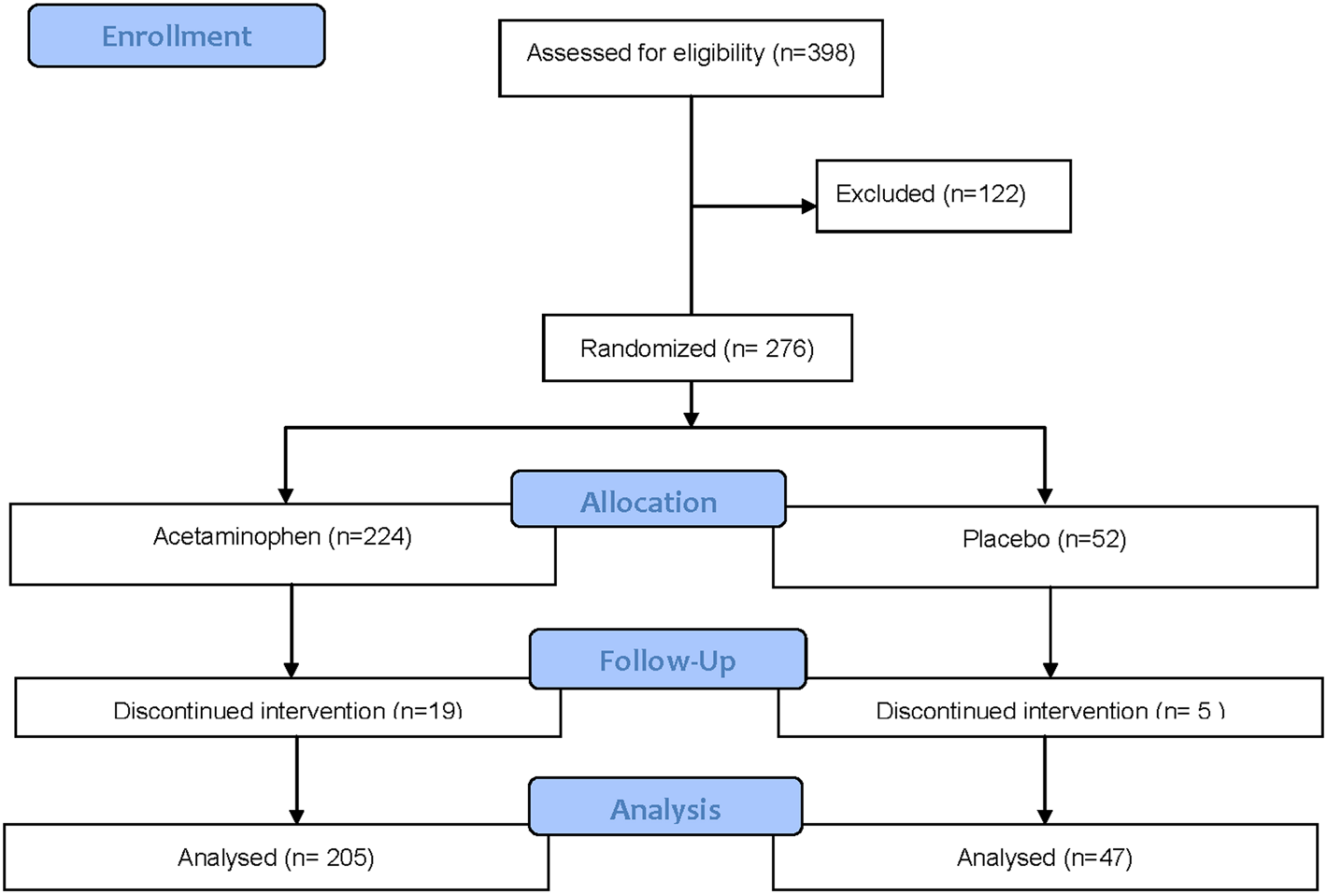
Consort diagram for study subjects.

**Figure 3:**
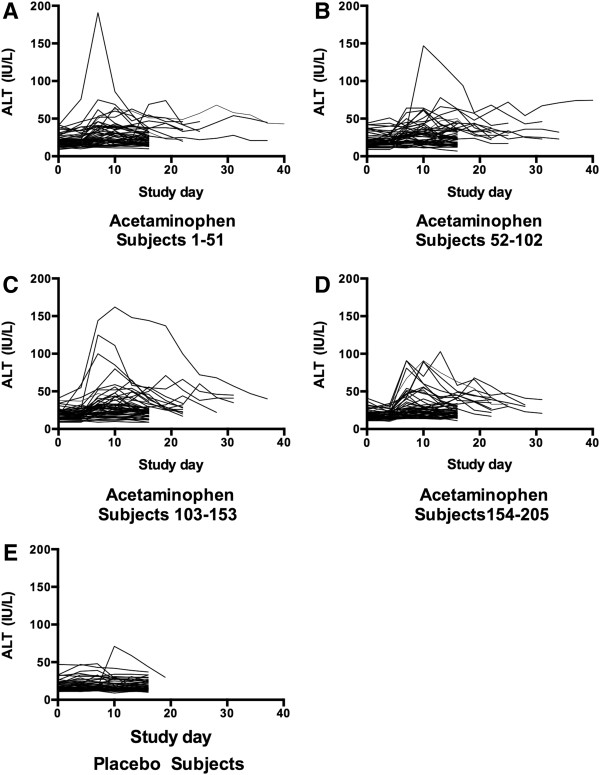
**Course of ALT for subjects administered acetaminophen (Panels A-D) and placebo (Panel E).** Acetaminophen subjects were split into 4 groups to allow better resolution of subject values.

**Figure 4:**
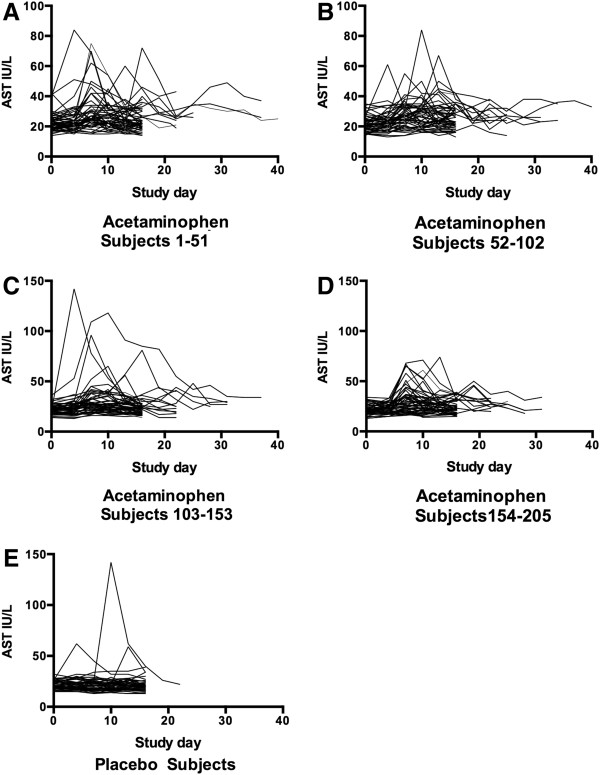
**Course of AST for subjects administered acetaminophen (Panels A-D) and placebo (Panel E).** Acetaminophen subjects were split into 4 groups to allow better resolution of subject values.

A total of 398 subjects were screened for the study, 122 were excluded at baseline, 276 were randomized and 252 completed the study. Of the 122 excluded patients, 109 (89.3%) did not meet eligibility criteria at screening and baseline and 13 withdrew participation prior to confirming full eligibility (5 withdrew consent, 5 did not return for baseline eligibility assessments, and 3 withdrew because they could not tolerate the venipuncture). The most common reasons for screen failure (subjects could fail more than 1 criteria) were elevated bilirubin (n = 47), elevated INR (n = 22), elevated ALT or AST (n = 28), elevated alkaline phosphatase (n = 4), positive viral hepatitis markers (n = 17), history of excessive alcohol consumption (n = 9), starting a new prescription in the preceding month (n = 4) and history of acetaminophen ingestion in the 3 days prior to enrollment (n = 3). The study was started on August 20, 2008 and stopped on August 16, 2011 at less than the planned number of subjects due to a higher than expected (31%) screen failure rate. Figure 2 is a CONSORT diagram showing the disposition of study subjects. Placebo and acetaminophen-treated subjects had similar baseline characteristics (Table 1).The median ALT in the acetaminophen group (Figure 3 panels A-D) was unchanged from baseline at study day 4, but increased by 6 IU/L to 26 IU/L on study day 7. The highest median ALT was observed on study day 10 (26.5 IU/L) and the median ALT decreased to 23.5 IU/L by study day 16. Changes in AST followed a slightly more accelerated course of elevation with the median increasing from 21 IU/L at baseline to 27 IU/L on day 7 and decreasing back to 23 IU/L on day 16 (Figure 4 panels A-D). While there was some day-to-day variation in subject measurements, the median ALT and AST in the placebo group was stable throughout the study period (Figures 3 and 4 panel E). The changes in ALT over time in the acetaminophen group were significant (p < 0.001 repeated measure ANOVA) and the day 16 ALT was higher for the acetaminophen than the placebo group (p < 0.001).

**Table 1:** Baseline characeteristics of study patients

	**Placebo (n = 47)**	**Acetaminophen (n = 205)**
**Age (years)**	32 (19.0, 64.0)	34 (18.0 to 74.0)
**Male**	12 (25.5%)	57 (27.8%)
**Race**		
**White**	35 (74.5%)	142 (69.3%)
** Hispanic**	4 (8.5%)	33 (16.1%)
** Black**	4 (8.5%)	13 (6.3%)
** Other/Mixed**	4 (8.5%)	17 (8.3%)
**Body Mass Index**	23.8 (17.3, 48.8)	25.8 (16.5, 53.5)
**Self-reported ethanol use**	35 (74.5%)	167 (81.5%)
**Baseline ALT**	17.0 (11.0, 47.0)	19.0(9.0, 44.0)

Note: median (range) were provided for Age, BMI and Baseline ALT.

Of the 205 acetaminophen subjects who completed the study, 157 (77%) did not have ALT elevations or had transient elevations that met resolution criteria by study day 16. Forty-eight completed subjects (23%) in the acetaminophen group and 1 subject (2%) in the placebo group had ALT elevation that met criteria at day 16 to enter the extended treatment phase. Of these 49 subjects, the majority (n = 31) entered the extended treatment phase because they had an ALT greater than10 IU/L above their baseline but less than or equal to the upper resolution criteria limit of 47 IU/L (including the one placebo subject) at study day 16. One subject had an ALT less than10 IU/L above their baseline but greater than 47 IU/L while 17 subjects had an ALT that was both greater than10 IU/L above their baseline and greater than 47 IU/L. Among the 49 subjects who entered the extended treatment period, 19 had a final ALT value at least 10 IU above the baseline ALT value.

**Figure 5:**
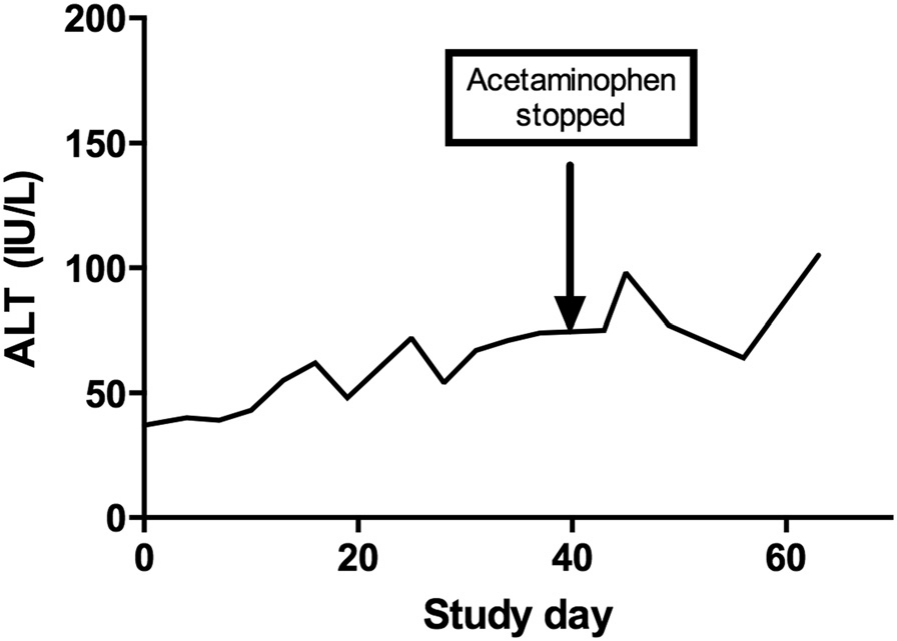
**Course of ALT for the one subject who did not have resolution of ALT elevation by study day 40.** Acetaminophen was stopped at study day 40 and ALT continued to elevate.

Overall, 204/205 (99.5%; 95% confidence interval 97.0 to 99.9%) acetaminophen subjects and 47/47 (100%, 95% confidence interval 91.0 to 100%) placebo subjects met criteria for resolution of ALT elevation by study day 40 or less while on continued study dosing regimen (acetaminophen or placebo). The median INR was 1.0 for the acetaminophen group at all time points through study day 36. The two subjects who had an INR measured at study day 40 had a median INR of 1.1. No patient in either group had liver dysfunction defined as elevation in INR above 1.4 or bilirubin greater than 2 times their baseline measurement. No subject had a serum acetaminophen concentration greater than 40 mcg/mL (264 micromol/L).The subject in the acetaminophen group who had an ALT elevation that did not resolve by day 40 was monitored for an additional three weeks after ending acetaminophen administration. After stopping study medication, her ALT continued to trend upward to a maximum of 105 IU/L on study Day 63 (Figure 5) and her INR and bilirubin remained normal. She had no symptoms of liver injury. She was referred for evaluation by a hepatologist but did not follow-up.

Among the completed subjects, 110 (54%) of acetaminophen subjects and 40 placebo subjects (85%) had a peak ALT less than 1.5 times their baseline while 95 acetaminophen subjects (46%) and 7 placebo subjects (15%) had an ALT greater than 1.5 times their baseline. Among the completed subjects, 115 (56%) acetaminophen subjects and 40 (85%) placebo subjects had a peak ALT < 10 U/L above baseline while 90 acetaminophen subjects (44%) and 7 placebo subjects (15%) had an ALT > 10 IU/L above their baseline. For each of these outcomes a logistic model was constructed to identify clinical or demographic characteristics associated with the outcome in the acetaminophen group. The independent factors in the model included demographics and baseline variables (age, sex ethnicity, baseline ALT, alcohol user, BMI) and study group. Age, sex, baseline ALT, alcohol use and BMI were not related to any outcome (Table 2). Ethnicity was not associated with entering the extended dosing outcome group. African American subjects were at increased risk for having an ALT increase more than 10 IU/L and there was an overall trend that non-Caucasian subjects had higher rates of all outcomes.

**Table 2:** Adjusted risk for secondary outcomes for subjects treated with 4 g/acetaminiophen/day

		**Extended dosing**		**ALT increase > 10 IU/L**		**ALT increase >50% above baseline**	
**Variable**		**Odds ratio**	**95% Confidence interval**	**Odds ratio**	**95% Confidence interval**	**Odds ratio**	**95% Confidence interval**
**Female (vs Male)**		1.47	0.64 to 3.73	1.03	0.53 to 2.03	1.21	0.61 to 2.39
**Race**							
** Caucasian**		1.0	Ref	1.0	Ref	1.0	Ref
** Black**		1.71	0.47 to 6.26	3.56	1.02 to 12.45	1.80	0.54 to 5.99
** Hispanic**		1.86	0.79 to 4.40	1.45	0.66 to 3.2	1.67	0.75 to 3.69
** Other**		1.85	0.58 to 5.91	1.40	0.50 to 3.96	1.81	0.63 to 5.24
**Age (per yr)**		1.01	0.98 to 1.04	1.02	0.99 to 1.04	1.02	1.00 to 1.05
**Baseline ALT (Per IU/L)**		1.03	0.98 to 1.08	1.01	0.97 to 1.05	0.96	0.92 to1.00
**BMI**		0.98	0.93 to 1.04	0.98	0.93 to 1.03	0.99	0.94 to 1.04
**No ethanol use**		1.81	0.81 to 4.1	0.90	0.42 to 1.92	0.89	0.42 to 1.88

A total of 19 acetaminophen subjects and 5 placebo subjects were withdrawn from the study after receiving at least one dose of study medication. Reasons for withdrawal included non-compliance (5 acetaminophen, 2 placebo), adverse event other than stopping criteria (4 acetaminophen, 1 placebo), day 0 ALT > 47 IU/L (4 acetaminophen) and subject request (6 acetaminophen, 2 placebo). The reasons subjects gave for requesting to be removed from the trial were: inconvenience/too much time required to participate (4 acetaminophen); no transportation (1 acetaminophen, 1 placebo); moved out of state (1 acetaminophen); and no reason given (1 placebo). No subject who requested withdrawal had any symptoms of liver injury. At the time of withdrawal, the median (range) ALT for acetaminophen subjects was 19.5 IU/L (17.0, 26.0 IU/L) for those withdrawn for non-compliance, 19 IU/L (15.0 to 28.0 IU/L) for those withdrawn after adverse events and 40.0 IU/L (13 to 120 IU/L) for those requesting withdrawal. Two subjects requesting withdrawal had an ALT above the reference range. One subject had a peak ALT of 120 on study day 10 that had decreased to 67 IU/L on day 28 when the subject stopped taking acetaminophen and withdrew. A follow-up ALT off medication on study day 30 was 54 IU/L. A second subject had an ALT of 36 on study day 16, stopped taking study medication on study day 18 and had an ALT of 119 IU/L on study day 21. His ALT decreased to 46 IU/L on study day 24 while not taking acetaminophen. For the placebo subjects requesting withdrawal, the ALT values at withdrawal were both 15 IU/L, for those removed for non-compliance the ALTs at the time of withdrawal were 11 and 21 IU/L and the subject removed for an adverse event had an ALT of 15 IU/L at the time of withdrawal. All subjects withdrawn had decreasing ALT documented on follow-up testing.

Self-reported medication compliance, as measured by the diary was similar for both groups. The median (interquartile range) percent of days where subjects took all 4 doses was 100% (86.7 to 100%) for the acetaminophen group and 86.5% (81.1 to 100%) for the placebo group.

### Adverse events

The overall proportion of subjects reporting any adverse event was 62.3%; 65.6% of the acetaminophen subjects and 48.1% of the placebo subjects. The characteristics of the adverse events are listed in the online supplement (Additional file 1: Table S3.pdf Adverse Events). The most commonly reported adverse events were gastrointestinal. The majority of adverse events were rated as unrelated or possibly related to study drug. Nausea was the most frequent adverse event rated as probably related or related to study drug. No serious adverse events were reported. The proportion of acetaminophen-treated subjects suffering adverse events was similar for those who had an ALT elevation >10 IU/L: nausea 15/90 (16.7%) in those with elevation and 19/115 (16.5%) in those without; vomiting 2/90 (2.2%) in those with elevation and 2/115 (1.7%) in those without and abdominal pain 6/90 (6.7%) in those with elevation and 6/115 (5.2%) in those without.

## Discussion

We found that ingestion of 4 grams per day of acetaminophen for a minimum of 16 days caused no significant change in ALT in approximately half of subjects, and a transient elevation that resolved by day 16 in approximately a quarter of patients. The remaining quarter of patients had modest ALT elevations that resolved when administration was continued over the next 24 days in all but one subject. The maximum observed ALT was 191 IU/L. No patient experienced drug-induced liver injury as defined by a Food and Drug Administration guideline despite continued therapy with acetaminophen while their aminotransferase activity was elevated. The proportion of acetaminophen subjects experiencing gastrointestinal symptoms was similar for those with and without ALT elevations.

Only one patient’s ALT elevation failed to resolve during continued administration of acetaminophen. The subject had no changes in INR or bilirubin, suggesting there was no liver dysfunction. Furthermore, this subject’s aminotransferase elevation continued to increase for 3 weeks after acetaminophen was stopped. As with any single case observation, it is not possible to determine causality, even within a clinical trial. However, this pattern of slowly progressive ALT elevation weeks after exposure is not consistent with acetaminophen-induced liver injury [15] and data from other randomized trials suggests that ALT elevations suspected to be related to acetaminophen resolve once acetaminophen is stopped [2,4,6,8]. This pattern of ALT increase suggests an alternative cause of aminotransferase elevation such as infection [16] or a change in diet [17]. Unfortunately, the subject declined a comprehensive hepatology evaluation (as planned in the study protocol). This limited our ability to identify potential causes for her ALT elevation.

As noted in previous studies [3,6], we found no association between aminotransferase elevation (using either of two definitions) and age or gender. While a previous study suggested an increased risk of elevation for Hispanic subjects [6], our study found no difference between Hispanic and Caucasian subjects. We also found no association between ethanol use and aminotransferase elevation. While there have been no previous trials that directly compare the effect of acetaminophen between drinkers and non-drinkers, separate studies have reported the effect sizes in these two groups. A study of moderate ethanol users administered 4 g/day of acetaminophen for 10 days reported a median ALT elevation of 8 IU/L [3] while a similar study in non-drinkers (using a more frequent sampling pattern) reported a median ALT elevation of 15 IU/L [4]. The current study suggests that the aminotransferase elevations do not differ between drinkers and non-drinkers and suggests that the differences in the previous trials are due to different sampling methods.

The degree of aminotransferase elevation caused by repeated therapeutic doses of acetaminophen has varied greatly among prior studies. Studies of ambulatory subjects have consistently reported that the highest observed elevation is less than 3 times the upper limit of normal [3-5,18], while studies of subjects confined to a clinical research facility reported elevations more than 8 times the upper limit of normal [2,6]. While the exact reason for these differences is not clear, there is meta-analytic evidence that subjects on a clinical research unit may increase their aminotransferase activity independent of treatment [19]. This meta-analysis compared the change in ALT for the placebo groups in studies where subjects were admitted to a clinical research unit to the change in ALT for the placebo group in studies where subjects were ambulatory. They noted that almost 10% of placebo treated patients on a clinical research unit had an increase in ALT that was at least 50% of the reference range width, while no outpatient subjects had elevations of this degree. It is possible that the marked increases observed in the acetaminophen group within studies using a clinical research unit are due to a potentiation of the acetaminophen effect by confinement. The clinical implications of an interaction between hospitalization and suggested acetaminophen-induced ALT elevations warrant further investigation. In a small observational study of inpatients (surgical and medical patients), acetaminophen users had an increased risk of developing an elevation of ALT compared to non-acetaminophen users [20]. While the elevations observed in this study were minimal, it is possible that acetaminophen-induced ALT elevations could progress to clinical liver injury if potentiated by other factors such as CYP 2E1 induction, malnutrition or oxidative stress. However, analysis of prospective trials that treated adult or pediatric patients with the therapeutic doses for a variety of medical conditions found no cases of acute liver failure [1,21].

Acetaminophen is frequently used in both combination and single ingredient products for prolonged periods. The rarity of alleged cases of hepatic injury from therapeutic doses is strong evidence that these doses are safe even though ALT elevations occur. Until recently, ALT elevations alone during pre-clinical studies were considered a signal that would often lead a company to stop seeking approval for candidate drugs. However, the recent guidelines emphasize the importance of changes in hepatic function (INR and bilirubin) in addition to elevations of aminotransferase activity [22]. These guidelines recognize that a large number of medications cause self-limited ALT elevations that are not a precursor to clinical liver injury and are not an indication to stop therapy.

Other medications that cause self-limited aminotransferase elevations include amiodarone, tacrine, heparin, isoniazid and the statins [23-27]. While liver failure has rarely been linked to isoniazid and amiodarone, there are no cases definitively caused by tacrine or heparin and the cases linked to the statins have been questioned [28]. These observations have led some to postulate that these transient elevations of the serum transaminase reflect hepatic adaptation [22]. While the mechanism is not clear hepatic adaptation may also occur with repeated acetaminophen dosing.

Our study population was limited to healthy volunteers, so it is possible that subjects with underlying liver disease or other medical conditions may have a different course of ALT activity during prolonged acetaminophen dosing. Our internal validity is limited by the loss of several subjects prior to completing the protocol. It is possible that these subjects would not have resolved prior to completing the study. This would lead us to underestimate the proportion of subjects who did not resolve. We were also limited by not reaching our planned sample size, and this decreased our overall study power. Our study is also limited by the use of two biomarkers (ALT and AST). It is possible that other biomarkers may produce different patterns of change.

## Conclusions

It is now clear that therapeutic doses of acetaminophen when administered for consecutive days may cause mild to moderate ALT elevations. These aminotransferase elevations are relatively common, usually less than 10 IU/L, not accompanied by liver dysfunction and resolve even when acetaminophen therapy is continued.

## Abbreviations

AST: Aspartate aminotransferase; ALT: Alanine aminotransferase; BMI: Body mass index; CI: Confidence interval; INR: International Normalized Ratiol; ULN: Upper limit of normal.

## Competing interests

This work was funded by an investigator-initiated award from McNeil Consumer Healthcare to Denver Health. The funding sponsor had no role in the conduct or analysis of the research. The manuscript was reviewed by the funding sponsor for proprietary information prior to publication, but the authors had the final decisions on all content. All authors were employees of Denver Health at the time of this study. Denver Health has research, consulting and clinical contracts with McNeil Consumer Healthcare. All authors received only their salary for work on this project. Dr. Heard was supported in part by Award Number K08DA020573 from the National Institute on Drug Abuse. The content is solely the responsibility of the authors and does not necessarily represent the official views of the National Institute on Drug Abuse or the National Institutes of Health.

## Authors’ contributions

KH, JLG, RCD designed the study, KH, VA and JLG performed the research, BBB analyzed the data, KH drafted the manuscript and all authors contributed substantially to the revisions. KH had full access to the data and assumes responsibility for the manuscript. All authors read and approved the final manuscript.

## Pre-publication history

The pre-publication history for this paper can be accessed here:

http://www.biomedcentral.com/2050-6511/15/39/prepub

## Supplementary Material

Additional file 1: Table S3 Adverse Events by class for all randomized subjects. Subjects were treated with acetaminophen (4 g/day) or placebo). Click here for file
