# Supplementary material for: A randomized, placebo-controlled trial to determine the course of aminotransferase elevation during prolonged acetaminophen administration
Source: BMC Pharmacol Toxicol. 2014 Jul 22;15:39. doi: 10.1186/2050-6511-15-39 (PMC4118644; doi:10.1186/2050-6511-15-39)
Supplement: Additional file 1: Table S3 — Adverse Events by class for all randomized subjects. Subjects were treated with acetaminophen (4 g/day) or placebo). [file 2050-6511-15-39-S1.pdf]

Supplemental Table 3. Adverse effects by class for all randomized subjects. Subjects were treated with acetaminophen (4g/day) or placebo).

System Organ Class	Acetaminophen Incidence n=224	Placebo Incidence n=52	Total Incidence n=276	p Value
Ear and labyrinth disorders	3 (1.3%)	0 (0.0%)	3 (1.1%)	1.0000
Eye disorders	2 (0.9%)	0 (0.0%)	2 (0.7%)	1.0000
Gastrointestinal disorders	70 (31.3%)	10 (19.2%)	80 (29.0%)	0.0922
General disorders and administration site conditions	23 (10.3%)	3 (5.8%)	26 (9.4%)	0.4331
Infections and infestations	14 (6.3%)	2 (3.8%)	16 (5.8%)	0.7443
Injury, poisoning and procedural complications	10 (4.5%)	2 (3.8%)	12 (4.3%)	1.0000
Metabolism and nutrition disorders	3 (1.3%)	1 (1.9%)	4 (1.4%)	0.5683
Musculoskeletal and connective tissue disorders	26 (11.6%)	5 (9.6%)	31 (11.2%)	0.8106
Nervous system disorders	42 (18.8%)	11 (21.2%)	53 (19.2%)	0.6978
Psychiatric disorders	6 (2.7%)	1 (1.9%)	7 (2.5%)	1.0000
Renal and urinary disorders	0 (0.0%)	1 (1.9%)	1 (0.4%)	0.1884
Reproductive system and breast disorders	5 (2.2%)	1 (1.9%)	6 (2.2%)	1.0000
Respiratory, thoracic and mediastinal disorders	23 (10.3%)	5 (9.6%)	28 (10.1%)	1.0000
Skin and subcutaneous tissue disorders	9 (4.0%)	0 (0.0%)	9 (3.3%)	0.2160
Vascular disorders	9 (4.0%)	1 (1.9%)	10 (3.6%)	0.6935
P value is based on Fisher's exact test.				
